# Pectin Hydrogels as Structural Platform for Antibacterial Drug Delivery

**DOI:** 10.3390/polym16223202

**Published:** 2024-11-19

**Authors:** Tejas Saravanan, Jennifer M. Pan, Franz G. Zingl, Matthew K. Waldor, Yifan Zheng, Hassan A. Khalil, Steven J. Mentzer

**Affiliations:** 1 Laboratory of Adaptive and Regenerative Biology, Brigham & Women’s Hospital, Harvard Medical School, Boston, MA 02115, USA; tjsdbh@umkc.edu (T.S.); jmpan@bidmc.harvard.edu (J.M.P.); yifan.a.zheng@gmail.com (Y.Z.); hakhalil@bwh.harvard.edu (H.A.K.); 2 Division of Infectious Diseases, Brigham & Women’s Hospital, Department of Microbiology, Harvard Medical School, Boston, MA 02115, USA; franz_zingl@hms.harvard.edu (F.G.Z.); mwaldor@bwh.harvard.edu (M.K.W.)

**Keywords:** high-methoxyl pectin (HMP), low-methoxyl pectin (LMP), work of cohesion (WoC), carbenicillin (CB), chloramphenicol (CHL), CMC, carboxymethylcellulose and kanamycin (KAN)

## Abstract

Hydrogels are hydrophilic 3-dimensional networks characterized by the retention of a large amount of water. Because of their water component, hydrogels are a promising method for targeted drug delivery. The water component, or “free volume”, is a potential vehicle for protein drugs. A particularly intriguing hydrogel is pectin. In addition to a generous free volume, pectin has structural characteristics that facilitate hydrogel binding to the glycocalyceal surface of visceral organs. To test drug function and pectin integrity after loading, we compared pectin films from four distinct plant sources: lemon, potato, soybean, and sugar beet. The pectin films were tested for their micromechanical properties and intrinsic antibacterial activity. Lemon pectin films demonstrated the greatest cohesion at 30% water content. Moreover, modest growth inhibition was observed with lemon pectin (*p* < 0.05). No effective inhibition was observed with soybean, potato, or sugar beet films (*p* > 0.05). In contrast, lemon pectin films embedded with carbenicillin, chloramphenicol, or kanamycin demonstrated significant bacterial growth inhibition (*p* < 0.05). The antibacterial activity was similar when the antibiotics were embedded in inert filter disks or pectin disks (*p* > 0.05). We conclude that lemon pectin films represent a promising structural platform for antibacterial drug delivery.

## 1. Introduction

Drug delivery is typically achieved through oral and intravenous administration. Because the drugs are not selectively targeted, systemic delivery typically requires repeated administration and high doses. Protein and peptide drugs are particularly inefficient, with serum half-lives ranging from minutes to hours [1]. A potential solution to inefficient administration is targeted drug delivery. In contrast to untargeted systemic administration, drug delivery targeted to specific sites or tissues provides the opportunity to increase local concentrations, improve efficacy, and limit toxicity.

Targeted drug delivery typically involves both a carrier system and a targeting mechanism. Carrier systems are designed to transport drugs without significant physicochemical modification or loss of function. Common drug carrier systems include liposomes [2], polymeric nanoparticles [3], micelles [4], and hydrogels [5]. To deliver the drug to a specific site or tissue, the carrier system requires a targeting mechanism. Common targeting mechanisms include antibodies, peptide ligands, small molecules, and aptamers [6,7].

We have previously shown that a hydrogel called pectin is a promising carrier system capable of selective drug delivery [8]. Pectin has a large water “free volume” that can serve as a delivery vehicle for soluble drugs. In addition, pectin is a plant-derived structural heteropolysaccharide that strongly binds to the glycocalyceal surface of visceral organs [9]. In addition to providing strong adhesion, the interaction of pectin with the glycocalyx facilitates the transport of tracer molecules across the glycocalyceal charge barrier [10]. Despite these insights, the effect of the pectin hydrogel on the function of the embedded drug is unknown. In this report, we compared pectin films from four distinct plant sources for their micromechanical properties and intrinsic antibacterial activity. Further, we used Kirby–Bauer testing to evaluate the functional activity of antibiotics embedded in the pectin hydrogel.

## 2. Methods

**Pectin.** The lemon pectin was supplied by a commercial source (Cargill, Minneapolis, MN, USA). The proportion of galacturonic acid residues in the methyl ester form determined the degree of methoxylation. The lemon pectin demonstrated a 50% degree of methoxylation. The pectins derived from potato (pectic galactan), soybean (rhamnogalacturonan), and sugar beet (arabinan) were obtained from Megazyme (Chicago, IL, USA). The pectin powder from all sources was stored under low humidity at 25 °C.

**Pectin dissolution in water.** The pectin powder was progressively dissolved at 25 °C to avoid undissolved powder [11]. The complete dissolution of the pectin was achieved by a high-shear 10,000 rpm rotor–stator mixer (L5M-A, Silverson, East Longmeadow, MA USA). The dissolved pectin was poured into custom molds prior to curing.

**Humidification chamber.** To minimize variation in ambient humidity, humidification was produced by an ultrasonic humidifier or within a custom 5.7-L chamber. The chamber was monitored by wireless (Bluetooth) hygrometer and thermometer sensors (Inkbird, Shenzhen, PRC). The custom system ensured stable humidification throughout each experiment.

**Colorimetric tracer.** Commonly called methylene blue, the diffusion tracer was methylthioninium chloride (Sigma-Aldrich, St. Louis, MO, USA). Methylene blue is a heterocyclic thiazine dye with the following molecular formula: C_16_H_18_C_l_N_3_S. Methylene blue was used as a standard 1% (*w*/*v*) solution.

**Spectrophotometry.** Spectrophotometry measurements have been described in detail elsewhere [12]. Briefly, the Minolta portable spectrophotometer (CM-508d, Minolta, Ramsey, NJ, USA) recorded colorimetric tracer diffusion within the agar. Wavelengths between 620 and 640 nm were used in the diffusion experiments. Replicate measurements were obtained with automatic averaging. The spectrophotometric data were processed (SpectraMagic, Minolta) and transferred to Microsoft Excel 365 for further analysis.

**Antibiotics.** All the antibiotics were obtained from commercial sources (Sigma-Aldrich) Carbenicillin CB is a semi-synthetic, broad-spectrum beta-lactam antibiotic derived from penicillin, characterized by its carboxybenzyl side chain, which enhances its activity against Gram-negative organisms, particularly Pseudomonas aeruginosa. Chloramphenicol (CMP) is a broad-spectrum antibiotic that binds to the 50S ribosomal subunit, blocking peptidyl transferase and halting peptide bond formation. Kanamycin (KAN) is an aminoglycoside antibiotic that exerts its bactericidal effect by irreversibly binding to the 30S subunit of bacterial ribosomes, leading to the misreading of mRNA and the disruption of protein synthesis. KAN is primarily effective against aerobic Gram-negative bacteria and some Gram-positive species.

**Active loading.** Active loading was performed as previously described [8]. After calibration, the material analyzer (TA-XT plus; Stable Micro Systems, Godalming, Surrey, UK) was used to actively load the sample. The drug or tracer sample was added to a 25 mm diameter acrylic disk with pectin mounted on the surface. After sample loading on the pectin, the loading probe descended at a selectable velocity (<5 mm/s) and compression force (>5 N). Data were acquired at 500 points.

**Filter paper.** The filter paper was medium-speed, 85 g per square meter (gsm), with a 10 um pore size (Eisco Labs, Honeoye Falls, NY, USA). The filter paper was punched to a 10 mm circular diameter.

**Fracture mechanics.** Fracture mechanics were performed as previously described [13]. Briefly, the pectin polymers were tested with a controlled uniaxial load normal to the plane of the pectin film. The fracture lobe was applied a 5 mm stainless steel spherical probe mounted to a TA-XT plus (Stable Micro Systems). The stainless steel probe compressed the biopolymers at a test speed of 2 mm/s until fracture. The fracture force, distance, and time were digitally recorded. 

**Contact angle.** Contact angle measurements were obtained after 5 uL droplets were placed in 3 random areas of the pectin film. The droplets were imaged with a custom imaging system orientated perpendicular to the plane of the film. An image series was acquired using MetaMorph 7.10 software (Molecular Devices, Downingtown, PA, USA) that facilitated the measurement of the 3-phase contact angle.

**Escherichia coli (*E. coli*) K-12.** The Mueller–Hinton agar plates were inoculated with *E. coli* K-12 [14]. K-12, obtained from the American Type Culture Collection (ATCC, Manassas, VA, USA), is a non-pathogenic strain extensively used in microbiology research. *E. coli* K-12 was used because of its well-characterized genome and ease of culture. 

**Kirby–Bauer test.** The Kirby–Bauer disk diffusion susceptibility test was performed using LB agar plates inoculated with 200 uL of bacterial cultures standardized to ~0.4 OD600 units. Antibiotic-impregnated filter disks or corresponding pectin disks were then placed on the inoculated agar using sterile forceps. The disks were sufficiently spaced to prevent overlapping zones of inhibition. The plates were incubated while inverted at 37 °C for 16–18 h. Post-incubation, the diameters of the inhibition zones around each disk were measured in millimeters. Proper agar depth and fresh materials were maintained to ensure reliable results.

**Statistical analysis.** Statistical analysis was performed on a minimum of 3 replicate samples. The unpaired Student’s *t* test for samples of unequal variances was used to calculate statistical significance. Where appropriate, the data were expressed as the mean + one standard deviation. The significance level for the sample distribution was set as *p* < 0.05.

## 3. Results

**Figure 1:**
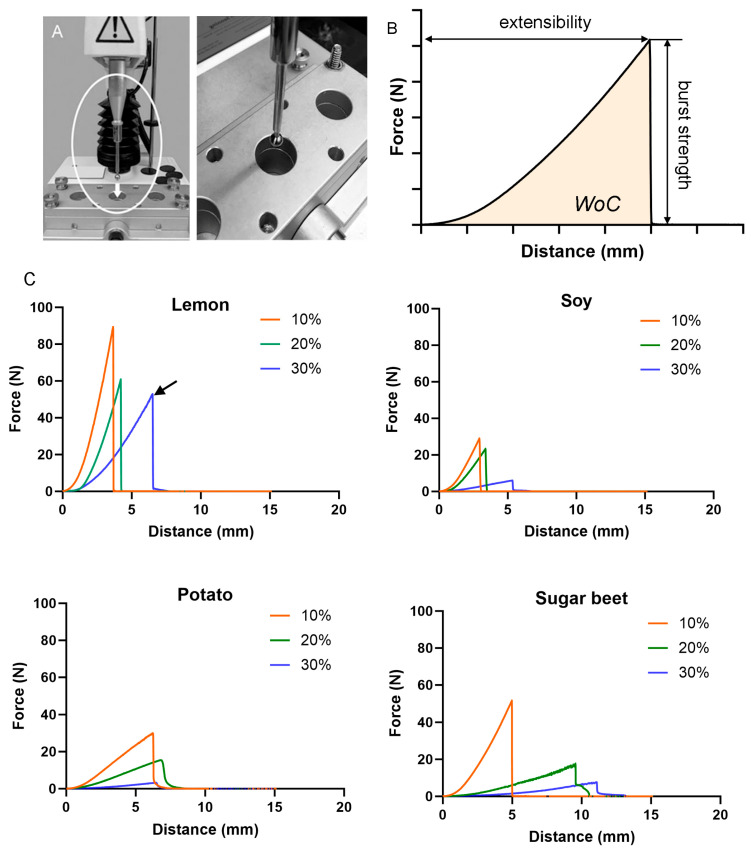
Burst strength and fracture mechanics of pectin films. (**A**) A load cell, mounted with a spherical stainless steel probe (5 mm), was used to assess film mechanics at various water contents. The pectin films were loaded until they fractured. (**B**) The peak force was recorded as the burst strength. The distance that the probe traveled was defined as the extensibility. The area under the force–displacement curve was defined as the work of cohesion (WoC). (**C**) Modal curves of seven replicates with 10%, 20%, and 30% water content are shown. Identical testing of pectin films derived from different plant sources—lemon, soybean, potato and sugar beet–are shown. Because of their superior micromechanics (arrow), lemon-derived pectin films were used in most experiments.

**Pectin micromechanics.** The pectin films were derived from four different plant sources: lemon, soybean, potato, and sugar beet. The micromechanical properties of the films were evaluated using a standard burst test (Figure 1A). The strength of cohesion and extensibility was assessed at water contents of 10%, 20%, and 30% (Figure 1B). For all plant sources, the pectin cohesive strength declined with increasing water content (Figure 1C). The lemon films demonstrated the greatest cohesion at 30% water content (arrow). 

**Figure 2:**
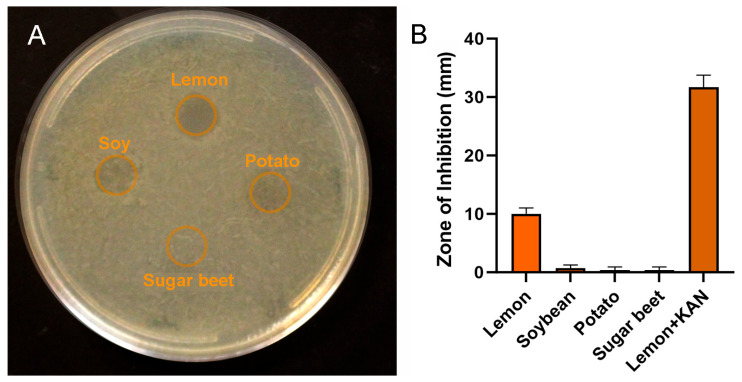
Antimicrobial properties of pectin films derived from four distinct plant sources: lemon, soybean, potato, and sugar beet. (**A**) Pectin disks (10 mm diameter) from each pectin source were applied to the agar plate inoculated with *E. coli*. The zone of inhibition was assessed at 18 to 24 h. (**B**) Modest bacterial growth inhibition was observed with the lemon pectin disk. For comparison, kanamycin was actively loaded into a lemon pectin disk (lemon + KAN). The mean diameter of the zone of inhibition ± 1 standard deviation is shown for replicate samples.

**Antimicrobial properties.** The pectin films derived from the four plant sources were evaluated for their intrinsic antimicrobial properties. The pectin film disks (10 mm) at 30% water content, without loaded drug, were placed on LB agar plates inoculated with Gram-negative bacteria (E. coli) (Figure 2A). Modest growth inhibition (10 mm diameter) was observed with lemon pectin (*p* < 0.05), but no effective inhibition was observed with soybean, potato, or sugar beet films (*p* > 0.05) (Figure 2B). Lemon pectin films loaded with kanamycin (lemon+) are shown for comparison (Figure 2B).

**Figure 3:**
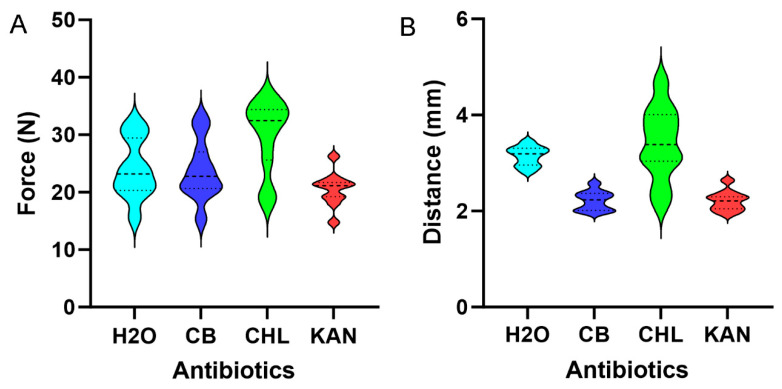
Effect of embedded antibiotics on lemon pectin micromechanics. (**A**) Burst strength was calculated for the pectin films loaded with water (H_2_O), carbenicillin (CB), chloramphenicol (CHL), and kanamycin (KAN). Notably, CHL resulted in a significant increase in burst strength (*p* < 0.05). (**B**) Similarly, extensibility was calculated for antibiotic-loaded lemon pectin films. Again, CHL had an unexpected effect on extensibility (*p* < 0.05). Median (dashed line) and quartile values (dotted lines) are shown in the violin plot.

**Antibiotic effects on pectin micromechanics.** Lemon pectin films loaded with water, CB, CHL, and KAN were assessed for burst strength and extensibility. CB and KAN had no significant effect on lemon pectin cohesion; that is, burst strength was not significantly different from water alone (*p* > 0.05). Unexpectedly, CHL significantly increased burst strength (*p* < 0.05) (Figure 3A). Similarly, CHL increased film extensibility (Figure 3B). To provide insights into the CHL effect on the lemon pectin films, we measured the contact angles of the three antibiotics interacting on the surface of the lemon pectin films. The droplet angles for CB (61 + 7) and KAN (72 + 5) were less than water 103 + 3 degrees, demonstrating moderate hydrophilicity and wettability. The rapid absorption of CHL precluded precise angle measurement; however, we estimated a CHL droplet contact angle of less than 20%.

**Figure 4:**
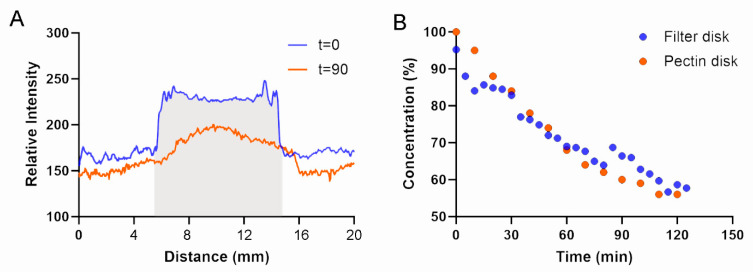
Tracer diffusion from filtered disks placed on agar plates. (**A**) To illustrate the assay, a filter disk loaded with 1% (*w*/*v*) methylene blue was placed on an agar plate, and diffusion was monitored by a color-thresholded optical linescan (MetaMorph) and confirmed with spectroscopy at 640 nm. The original boundary of the pectin disk is shown in light gray. (**B**) The methylene blue concentration on the agar plates was estimated based on standard curves (not shown). The rate of methylene blue diffusion from the filter disks and pectin disks reached a plateau between 2 and 5 h.

**Figure 5:**
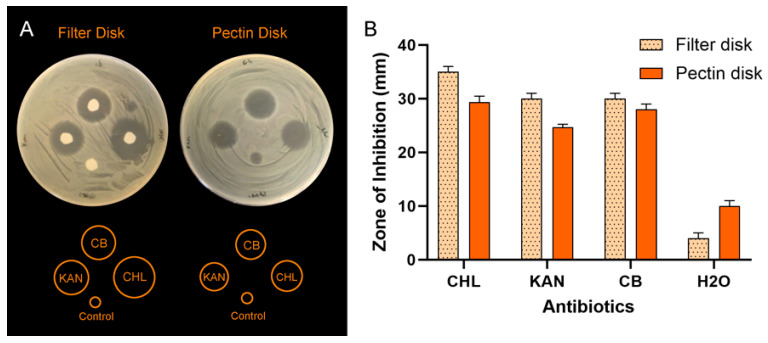
Comparison of bacterial growth inhibition of antibiotics loaded into filter disks and pectin disks. (**A**) Representative example of growth plates treated by antibiotics loaded into filter disks and pectin disks. The corresponding annotation is shown below the plate. (**B**) Replicate samples were reproducible. CHL and KAN demonstrated a modest but significant increase in growth inhibition when loaded into the filter disk (*p* < 0.05). Error bars indicate mean ± 1 standard deviation.

**Disk diffusion.** Both filter disks and pectin disks rely upon antibiotic diffusion through the disk pores. To compare the diffusion characteristics of filter and pectin disks, we loaded both 10 mm disks with the tracer methylene blue. The diffusion characteristics of tracer-loaded filter and pectin disks were virtually identical at t = 0 with a similar diffusion pattern at t = 90 min (Figure 4A). For both tracer disks, the initial diffusion plateau was identified at 2 h (Figure 4B), with a definitive plateau at 5 h. Consistent with similar diffusion characteristics, antibiotics were loaded into the filter disks, and pectin disks demonstrated similar bacterial growth inhibition (Figure 5).

## 4. Discussion

Here, we investigated the suitability of pectin hydrogels, derived from four different plant sources, to serve as a structural platform for antibacterial drug delivery. There were several relevant findings. First, lemon was the only pectin source that demonstrated intrinsic antibacterial activity. Soybean, potato, and sugar beet sources demonstrated no measurable antibacterial activity. Second, lemon pectin films were structurally superior to other plant sources at higher water contents. Lemon pectin hydrogels demonstrated a greater burst strength and work of cohesion. Third, loaded lemon pectin films delivered antibiotics with preserved antibacterial activity. Similarly sized pectin disks and filter disks loaded with KAN, CHL, and CB demonstrated comparable zones of inhibition. Fourth, CHL-loaded pectin films demonstrated altered micromechanics. This poorly understood feature of CHL may reflect its hydrophilic character and nitrobenzene structure. We conclude that lemon pectin films represent a promising structural platform for antibacterial drug delivery to the surface of visceral organs.

Pectin is a complex structural polysaccharide present in plant cell walls. Its structure varies significantly among different plant sources. Because of these structural differences, we studied pectins derived from lemon, potato, soybean, and sugar beet plants. The finding that lemon pectin demonstrated desirable micromechanical properties was not unexpected. Derived from lemon, orange, and apple sources, citrus pectin is characterized by a high degree of cohesion and adhesivity [9]. Pectin’s physicochemical characteristics vary with the degree of esterification [13]. High-methoxyl (HM) pectins have distinctive gel-forming characteristics, while low-methoxyl (LM) pectins gel in the presence of divalent cations like calcium ions [15,16]. Potato pectin, on the other hand, has a lower degree of esterification and a higher content of neutral sugars, which produces a less rigid gel structure. Soybean pectin is unique due to its higher protein content and lower molecular weight, which affect its gelling properties. Sugar beet pectin is noted for its relatively high acetylation, which influences its solubility and weaker gelling behavior. In these experiments, we used CMC to facilitate gel formation in the sugar beet-derived pectin.

As a tissue adhesive that strongly binds to the surface of visceral organs [17,18], pectin films provide a promising method for targeted drug delivery. Structurally, pectin has a large water-containing “free volume.” The water component can serve as a vehicle without the unintended chemical modification of the hydrogel polymer or the embedded drug. Here, the lemon pectin films were structurally superior to other plant-derived films, maintaining their cohesion and extensibility despite active loading with CB, CHL, or KAN.

Active loading involves the use of external forces to incorporate the drug into the hydrogel. An advantage of active loading is the ability to precisely control the amount and distribution of the drug within the pectin films. Passive loading, in contrast, relies upon the spontaneous absorption and diffusion of the drug into the pectin film. Passive loading is less well controlled, with loaded concentrations dependent upon relevant surface energies and evaporative losses. Of note, the dramatic hydrophilicity of CHL suggests that the method of drug loading is a relevant consideration for future work.

The zone of inhibition in the Kirby–Bauer test depends upon the effective diffusion (drug release) of the antibiotic out of the filter disk or pectin disk. Specifically, if the polymer pores are larger than the drug molecules, the release rate is controlled by the steric interactions between the drug and the polymer network. Conversely, if the pores are smaller or comparable in size to the drug molecules, then the release rate is slower. In our experiments, the filter disk had a known pore size of 10 um; the pore size of the pectin disk was estimated by tracer diffusion. When both filter disks and pectin disks were loaded with the tracer methylene blue, the zone of diffusion was similar. With both disks, most of the expected diffusion occurred within 90 min, with a final concentration plateau at 5 h.

A curious observation concerned the effect of CHL on pectin micromechanics. CHL significantly increased pectin cohesion (burst strength) and extensibility. These effects may reflect the unique properties of CHL. The chemical structure of CHL, C_11_H_12_C_l2_N_2_O_5_, reflects a unique nitrobenzene moiety that distinguishes it from other antibiotics. Since pectin cohesion is likely a reflection of hydrogen bonding and hydrophobic interactions between pectin chains, we speculate that CHL facilitated these interactions—perhaps by functioning as a crosslinker between chains [19]. The effect of CHL on pectin structure is an intriguing area for future work.

Finally, our work highlights the multi-functional potential of pectin polysaccharide. We have shown that pectin films strongly bind to the surface glycocalyx of visceral organs with a mechanism of branched chain entanglement [9,20,21]. The strong adhesivity has led to a proposed function of pectin films as a sealant for visceral organ injuries. Pectin has demonstrated the ability to seal pleural air leaks in the lung [21] and injured serosa in the gut [22]. Here, we show that pectin free volume can also contribute to targeted drug delivery. The implication of the present work is that water-soluble drugs can now be delivered directly to the surface of injured visceral organs without significant modification. Based on our findings with antibiotics, we anticipate that pectin films will be a useful delivery mechanism for a range of potential treatments, including coagulation factors, anti-inflammatories, and growth factors.

## Data Availability

Data available on request.
